# Bone Turnover Markers in Chronic Periodontitis: A Literature Review

**DOI:** 10.7759/cureus.6699

**Published:** 2020-01-19

**Authors:** Hamda Shazam, Fouzia Shaikh, Zaheer Hussain

**Affiliations:** 1 Department of Oral Pathology, Ziauddin College of Dentistry, Ziauddin University, Karachi, PAK; 2 Department of Pathology, Ziauddin University, Karachi, PAK; 3 Department of Periodontology, Altamash Institute of Dental Medicine, Karachi, PAK

**Keywords:** bone turnover biomarkers, chronic periodontitis, oral-fluid diagnostics

## Abstract

Chronic periodontitis (CP) is a multifactorial oral inflammatory disease characterized by progressive destruction of bone and ultimate tooth loss. The alarming rise in the prevalence of periodontitis has led to the development of innovative diagnostic techniques. Several quantifiable biomarkers in the gingival crevicular fluid (GCF) and saliva of chronic periodontitis patients have been detected in the field of oral fluid diagnostics. Bone turnover biomarkers hold a valuable diagnostic potential in determining the extent of alveolar bone destruction and the risk of future bone loss. This review article highlights the importance of bone turnover markers in facilitating earlier detection, accurate diagnosis, and effective treatment strategies, leading to optimal clinical management of chronic periodontitis.

## Introduction and background

This review article aims at providing a detailed outline of the role played by bone turnover markers in chronic periodontitis (CP). For this purpose, we searched for electronic databases involving Science Direct, PubMed, Scopus, and Google Scholar. A combination of related keywords was used such as saliva and bone turnover biomarkers, oral diagnostics and periodontitis, diagnosis of chronic periodontitis, and salivary biomarkers. Full-text, relevant articles published from 2001 to 2019 in dental journals, including case-control studies, cross-sectional studies, and systematic and short reviews were reviewed.

Periodontal disease (PD) is a set of infectious oral inflammatory conditions that affect the periodontal apparatus of the tooth. It results due to the disruption of the symbiotic relationship between oral flora and the host immune system, characterized by successive periods of microbial exacerbation followed by periods of remission, causing progressive tooth destruction and loss [1]. Generally, periodontal diseases are categorized into two groups, namely, gingivitis, an acute and reversible inflammation confined to the gingival tissues, which, if left untreated, leads to a more advanced, irreversible, and destructive form known as periodontitis [2]. The clinical manifestations of periodontitis include the formation of deep periodontal pockets, loss of periodontal ligament and cementum attachment, and resorption of the alveolar bone, which leads to ultimate tooth loss [3].

Periodontal disease is a major oral health burden, and according to the National Health Survey conducted in the UK, it affects 20%-50 % of the adult population globally. Chronic periodontitis, the more prevalent form of periodontal disease, accounts for affecting nearly 5%-15% of the adult population worldwide [4-5]. Its severity increases with advancing age, with a peak incidence of around 30-45 years of age [6]. The prevalence of chronic periodontitis varies according to different geographic regions and is seen to be the highest in Asian countries (about 15%-20%) [7]. In Pakistan, the prevalence of chronic periodontitis is reported to be 31.4% [8].

Individuals with chronic periodontitis may also encounter certain other negative impacts on their quality of life such as impaired mastication and swallowing, speech difficulties, esthetic concerns, and so on [9]. Chronic periodontitis has also been implicated in various systemic diseases such as cardiovascular disorders, renal abnormalities, diabetes mellitus, asthma, adverse birth outcomes, and obesity [4,10-11].

The etiology of chronic periodontitis is multifactorial. It is estimated that more than 700 bacterial species colonize the oral cavity, out of which nearly 400 species are present in subgingival areas. Three bacterial microorganisms, in particular, Aggregatibacter actinomycetecomitans, Tannerella forsythia, and Porphyromonas gingivalis are known to be the primary causative microbial agents [12-13]. Certain other environmental and acquired risk factors also attribute to the pathogenesis of periodontitis, which includes stress, poor oral hygiene, inadequate dietary habits, alcohol and tobacco consumption, smoking, and genetic factors [14]. The pathogenesis of chronic periodontitis involves the cascade of sequential events leading to host immunomodulatory responses triggered by toxic byproducts released from bacterial microbes. These bacterial byproducts, in turn, activate various cytokines, chemokines, pro-inflammatory mediators, and macrophages, which are responsible for the progressive destruction of underlying gingival tissues and subsequent tooth loss [15-16].

## Review

Role of oral fluid biomarkers in chronic periodontitis

**Figure 1:**
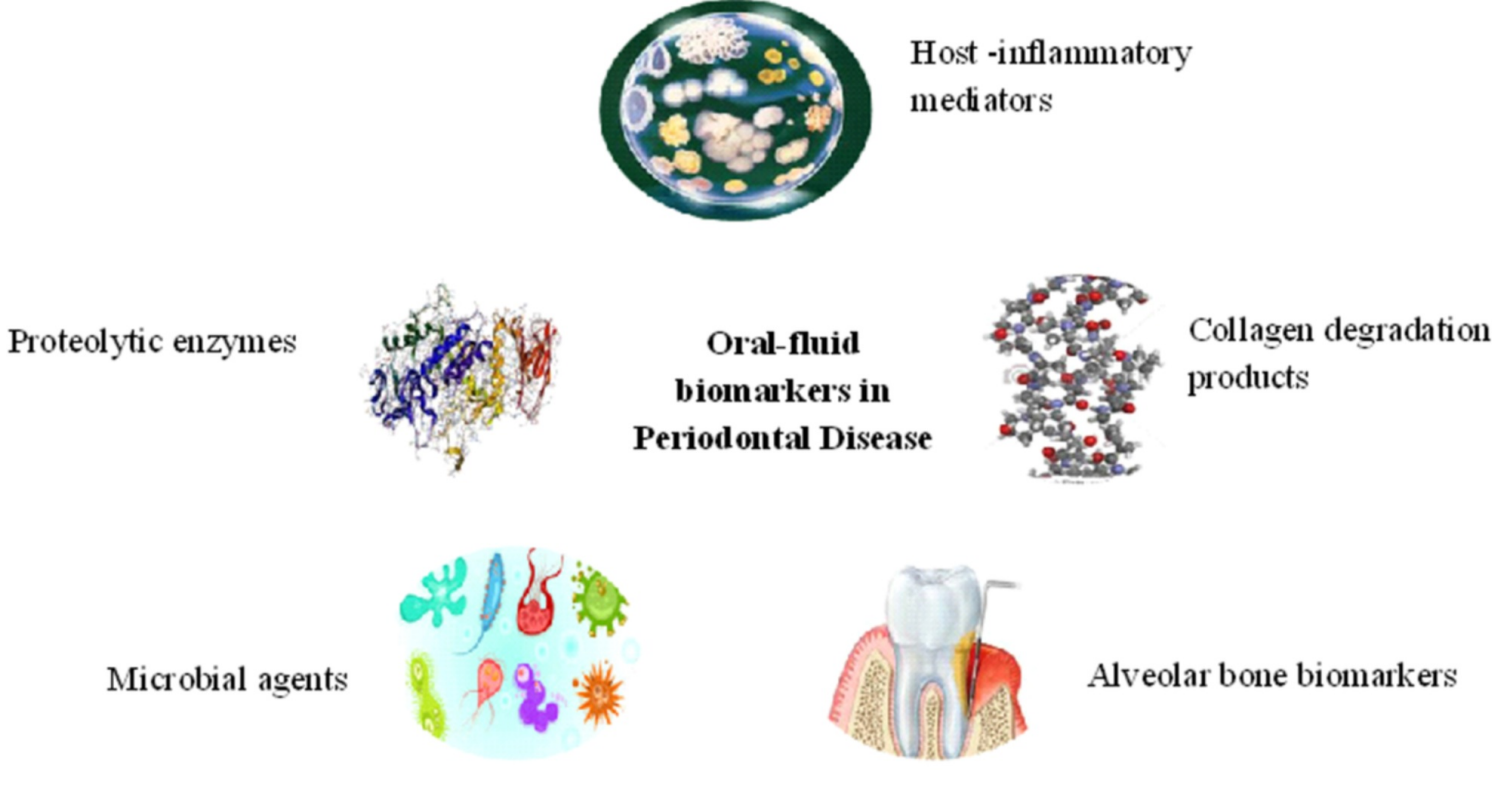
Types of oral fluid biomarkers in periodontal disease

Conventionally used diagnostic techniques to assess periodontitis include clinical and radiographic measurements. Commonly used clinical parameters are probing pocket depth (PPD), bleeding on probing (BOP), and clinical attachment loss (CAL). These methods are inherently limited only to a historical perspective and lack to provide pertinent knowledge about current disease status and activity. Apart from this, these techniques also lack to identify the highly susceptible individuals who are at greater risk of future bone loss [17]. Since the last few decades’ considerable research has been done in the field of oral fluid diagnostics, which detected various biomarkers in oral fluids such as saliva and gingival crevicular fluid (GCF) of periodontitis patients [18-19].These biomarkers include enzymes, proteins, hormones, host-derived biomolecules, deoxyribonucleic acid (DNA), ribonucleic acid (RNA), bacterial, and volatile products (Figure 1).

Due to their easily accessible and noninvasive nature, these biomarkers prove to be optimal diagnostic tools by providing earlier detection, better diagnosis, and timely management of chronic periodontitis [20]. In terms of definition, a biomarker is a quantitative variable that can be measured and evaluated as an indicator of normal biological processes, pathological processes, or pharmacological responses to a therapeutic intervention [21].

Bone turnover biomarkers in chronic periodontitis

Biomarkers of periodontal disease are often represented as molecules related to three pathological phases, i.e. inflammation, collagen degradation, and alveolar bone turn over. Bone is constantly undergoing the process of bone remodeling, which is an inevitable process in which the rate of bone deposition is faster than the rate of bone resorption to maintain bone hemostasis. However, in pathological conditions, such as periodontitis, this equilibrium between both rates is reversed and is reflected as an overall change in the alveolar bone turnover rate. The bone turnover rate is assessed by bone turnover markers (BTMs), which are further divided into bone formation markers and bone resorption markers. Alveolar bone loss is a critical aspect of periodontitis and, in light of this fact, different studies have quantified levels of bone turnover biomarkers in the GCF and saliva of periodontitis patients that were closely parallel to disease progression and severity [22-25]. Table 1 lists the different bone turnover biomarkers in periodontitis.

**Table 1:** Bone turnover biomarkers in periodontitis GCF = Gingival crevicular fluid; RANKL = Receptor activator of nuclear factor kappa-B ligand; PD = Periodontal disease

Biomarker	Role in Periodontitis	Detection medium
Bone formation markers		
Alkaline phosphatase (ALP)	Vital role in the calcification process and increases with PD severity	GCF, saliva
Osteocalcin	Stimulates bone formation and potential diagnostic bone turnover marker in periodontitis	GCF, saliva
C-terminal propeptide of type I procollagen (PICP)	Induces proliferation and differentiation of osteoblasts and fibroblasts	Saliva, GCF
Osteoprotegerin	Inhibits osteoclastic activity by binding to RANKL and increased in disease progression	Saliva, GCF
Bone resorption markers		
Cross-linked C-terminal of type I collagen(ICTP)	Actively participates in collagen degradation and increases in periodontitis	GCF, saliva
Cross-linked N-terminal telopeptide of type I collagen (fragments NTX)	Promotes bone resorption and increases in aggressive periodontitis	Saliva, GCF
Bone sialoprotein (BSP)	Increased osteoclastic activity	Saliva
RANKL	Responsible for increased osteoclastic activity during periodontitis	Saliva, GCF

Alkaline phosphatase

Alkaline phosphatase (ALP), a glycoprotein and hydrolase enzyme, is predominantly present in the renal, hepatic, and osteogenic cells. It hydrolyzes ester bonds at an alkaline pH due to which serum/plasma levels of phosphate ions are raised. In the periodontium, ALP plays a vital role in cementogenesis and the maintenance of bone homeostasis. It stimulates the calcification process and actively participates in the bone remodeling phase [26]. Evidence suggests that there exists a significant positive correlation between gingivitis, PPD, and elevated ALP levels in periodontal disease [27]. Koss et al. conducted a case-control study to investigate the levels of ALP among normal and periodontitis patients. In this study, it was observed that levels of ALP were increased in the saliva of periodontitis patients as compared to controls [28]. Hence, it might be concluded that ALP is a possible bone turnover marker, indicating the progression and severity of the periodontal disease.

Osteoprotegerin and RANKL

Osteoprotegerin (OPG) is another alveolar bone turnover marker that plays an essential role in the process of osteogenesis and bone hemostasis. It is also a glycoprotein having a high affinity for RANKL and suppresses its activity by inhibiting osteoclasts formation. RANKL is a cytokine and acts as a gene regulator and ligand for the receptor RANK that controls the activation, proliferation, and differentiation of osteoclasts, resulting in alveolar bone resorption. Several studies demonstrate the importance of the RANKL/OPG ratio in determining the pathogenesis and severity of periodontal disease [29]. In a cross-sectional study, the levels of OPG and RANKL were analyzed in the saliva of chronic periodontitis patients and it was found that gingival index (GI) (p=0.024), PPD (p<0.001) and clinical attachment loss (CAL) (p=0.002) were significantly correlated with RANKL/OPG ratio. All the clinical periodontal parameters were also significantly correlated with RANKL levels (p<0.05) [30]. LS Branco et al. investigated GCF samples of aggressive periodontitis patients and found PPD and CAL to be significantly correlated with RANKL [31]. Similarly, in another study done by Ochanji et al., the values of the RANKL/OPG ratio were positively correlated with periodontal disease severity [32]. Consistent with these results, the RANKL/OPG ratio should be considered as a putative diagnostic tool in evaluating periodontal disease.

ICTP

ICTP, also known as carboxyterminal telopeptide of type I collagen or pyridinoline, is a specific indicator of alveolar bone destruction in aggressive periodontitis patients. During periodontal disease progression, the levels of ICTP are directly proportional to increased collagen degradation. Mishra et al. evaluated the salivary levels of ICTP in patients of chronic periodontitis and gingivitis and the results depicted that ICTP levels were much higher in patients of periodontitis (p=0.001) than gingivitis [23]. Increased GCF levels of ICTP were also seen in chronic periodontitis patients with a statistically significant p-value (p=0.001) [33]. Therefore, ICTP is suggested to be a candidate marker in the prediction of future alveolar bone loss.

NTX

Cross-linked N-terminal telopeptide Type I collagen (NTx) is a short telopeptide, which is released as a byproduct of bone resorption. Several studies suggested that increased NTx levels in GCF may serve as predictive markers of alveolar bone destruction. GCF Levels of NTx were elevated in chronic periodontitis patients and positively correlated with clinical periodontal parameters [34-35].

Osteocalcin

Osteocalcin is the most abundant protein found in the extracellular matrix of bone. It contains glutamic acid residues and is synthesized by bone and cartilage cells. It actively binds to the Ca++ ions present in the hydroxyapatite crystals lattice of bone. In serum/plasma, it freely moves as the decarboxylated form, whereas it is present in bone as the inactive carboxylated form [36]. Osteocalcin is a marker of bone formation but due to its role in recruiting osteoclasts to the site of bone resorption, it is now widely accepted as a vital bone turnover marker [37]. Raised levels of osteocalcin in serum are associated with metabolic diseases such as rheumatoid arthritis, multiple myeloma, and bone regeneration. In a case-control study by Bullon et al., the levels of osteocalcin were examined in three different oral fluids i.e. GCF, serum, and saliva. Serum and saliva osteocalcin concentrations were not statistically different, however, osteocalcin concentrations in GCF were significantly elevated (p<0.008) [38]. The salivary levels of osteocalcin were significantly correlated with the clinical attachment level. Similarly, several other observations also revealed high levels of osteocalcin in the saliva of chronic periodontitis patients. Conclusively, it could be stated that osteocalcin not only holds a significant diagnostic potential but also can be used as a prognostic marker to predict the likely outcome of the disease [29,39-41].

Osteonectin

Osteonectin (SPARC) is a protein that is rich in cysteine. It is secreted by osteoblasts, fibroblasts, and red blood cells (RBCs) and is chemoattractant to calcium ions present in bone. Its levels in bone matrix commensurate with the bone formation process during bone remodeling. It upregulates bone formation by accelerating the differentiation of immature osteocytes to mature osteoblasts and recruit them to an active bone deposition site. Hence, osteonectin is considered as a predictive marker of bone formation [42]. M Baeza et al. analyzed levels of osteonectin in GCF samples of 106 chronic periodontitis patients and found significantly raised concentrations of osteonectin (p<0.05) [43]. Therefore, osteonectin is a bone-regulating protein and maintains periodontal ligament (PDL) hemostasis during the bone remodeling phase [44].

Osteopontin

Osteopontin is a glycoprotein found in non-mineralized tissues, primarily renal, endothelial cells, and epithelial cells. Osteopontin plays a dual function both in bone mineralization and bone resorption [45]. Osteopontin enhances dentine formation during the bone remodeling process and has been implicated in the recruitment of polymorphonuclear cells in response to infections [46]. Kido et al. reported in his case-control study that increased levels of GCF osteopontin coincided with PPD values and may be a possible marker of bone resorption [47]. In another study, GCF osteopontin levels were markedly raised in disease severity and reduced after six to eight weeks of nonsurgical treatment given to chronic periodontitis patients [48].

Calcium

Many studies supported the theory that increased levels of Ca++ ions in saliva develop a higher risk of periodontitis. Individuals at a higher risk are more prone to develop periodontitis due to alkaline oral pH that favors increased mineralization and elevated levels of calcium in saliva [49]. In a study, a positive correlation was observed between the salivary levels of calcium and clinical attachment loss [50].

## Conclusions

In this revolutionary era of oral-fluid based diagnostics, biomarkers are opted as putative diagnostic tools for better monitoring, diagnosis, and clinical management of periodontitis. In context to this, various quantitative and qualitative diagnostic approaches, including genomic profiling, proteomic analysis, and transcriptomics, have helped researches best define human physiology and pathology based on comprehensive screening of these biomarkers. The detection of bone biomarkers in the GCF or saliva of periodontitis patients provides information regarding the extent of alveolar bone involvement and predict susceptible individuals at risk of bone loss. Hence, these biomarkers not only aid in the detection of periodontal disease at an earlier stage but also reduce disease severity and progression. However, it is evident that additional longitudinal and observational studies are required to target the prognostic value and diagnostic accuracy of these bone turnover markers in periodontitis.
